# Data analysis and study of the influence of deposition power on the microstructural evolution and functionality of metallic phase composite coating

**DOI:** 10.1016/j.dib.2018.02.007

**Published:** 2018-02-07

**Authors:** T. Monyai, O.S.I. Fayomi, A.P.I. Popoola

**Affiliations:** a Department of Chemical, Metallurgical and Materials Engineering, Tshwane University of Technology, P.M.B. X680 Pretoria, South Africa; b Department of Mechanical Engineering, Covenant University, P.M.B. 1023 Ota, Nigeria

**Keywords:** Zn–Ni, Passive film, Mild steel, NbO_2_

## Abstract

In anticipation for resolution of deterioration catastrophe on metallic materials, researches in the field of corrosion remains. Zn–Ni–NbO_2_ deposits were obtained on mild steel substrate using D.C. power source. The thermal stability properties of the coatings were determined by micro-hardness evaluations before and after heat treatment at 250 and 350 °C. The surface structure analysis was done by Scanning Electron Microscope and X-ray diffraction while the wear evaluations were obtained and compared. The weight gain and coating thickness were obtained and found to be in correlation with the wear results. The coating developed in this study is recommended for metallic surface improvement engineering applications.

**Table:** 

Subject area	*Materials Engineering*
More specific subject area	*Surface Science and Engineering*
Type of data	*Table, Figures*
How data was acquired	Electrodeposition process from an electrolyte bath containing the NbO_2_ enhancing particle was done at a temperature of 35 °C. Prior to deposition the samples were mechanically and chemically prepared. The deposition voltage was varied between 0.5 and 10 V. The post plating analysis was done revealing the morphology structures through SEM/EDS and XRD. The effect of high temperature was explored at temperatures between 250 and 350 °C, average microhardness evaluations were utilised as stability indicators.
Data format	Raw, Analyzed
Experimental factors	Calibrated equipment was used in the process of obtaining the results to ensure precise and correct results data.
Experimental features	The deposited coatings were obtained from an electrolyte connected to DC power at 0.5 and 1.0 V for 20 min at a controlled temperature of 35 °C.The influence of the change in deposition applied voltage was investigated along with the additive composition variation.
Data source location	Department of Chemical, Metallurgical and Materials Engineering, Tshwane University of Technology, Pretoria, South Africa and Mechanical Engineering, Covenant University, Ota Ogun State, Nigeria
Data accessibility	Data are available within this article

**Value of the data**

• The resulting data will be useful for materials engineers by guiding them on the reaction of the formulated deposition electrolyte for a specific temperature application.
• The obtained data can be used to report on the relationship between the different variable in the study.
• The data is useful in providing a useful range of additive concentration enough for the improvement of the substrate material

## 1 Data

The depositions process was performed at 20 min at a voltage power supply variations between 0.5 and 1.0 V with temperature of 35 °C. The distance between the anode and cathode was kept constant while the NbO_2_ enhancing additive composition was varies between a concentration of 10 and 15 wt%. The data for the formulation of bath framework is presented in Table 1. The coating thickness, weight gained, coating per unit area data were obtained after weighing the final mass and gauging the coating thickness. A set of data were obtained from XRD analysis and the plotted into a graph while the microstructures results were generated directly from the PC connected to the SEM-EDS machine. The average microhardness data were obtained from five points for 15 s dwell with 100 g of load. Results show a good progression of coating strengthening effect of particulate with response to its process parameter.

**Table 1:** Summarized bath formulation (Zn–Ni–NbO_2_ deposition).

**Composition**	**Mass concentration (g/l)**
ZnSO_4_	150
Na_2_SO_4_	10
H_3_BO_3_	20
(NH_4_)_2_SO_4_	20
Glycine	10
Nickel powder	60
NbO_2_	10–15

**Constant parameters**	
pH	5
Voltage	0.5 V and 1.0 V
Time	20 min
Temperature	35 °C

## 2 Experimental design, materials and methods

**Fig. 1:**
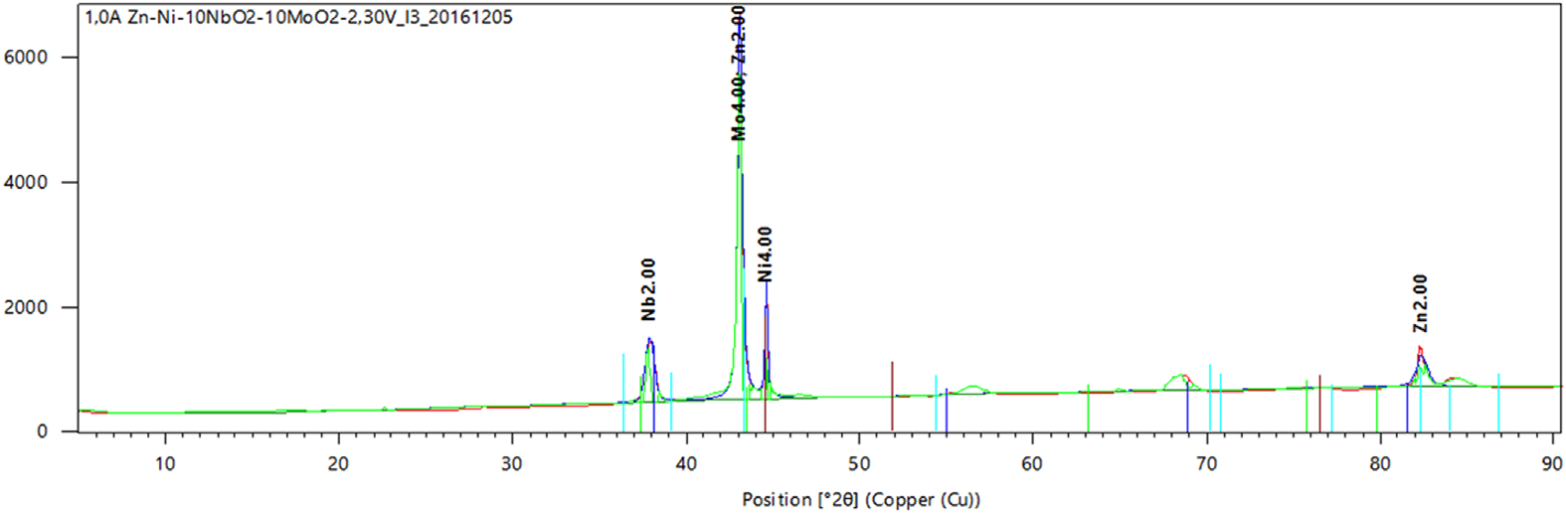
Solid x-ray diffraction profile for Zn–Ni–10NbO_2_–1.0V alloy.

**Fig. 2:**
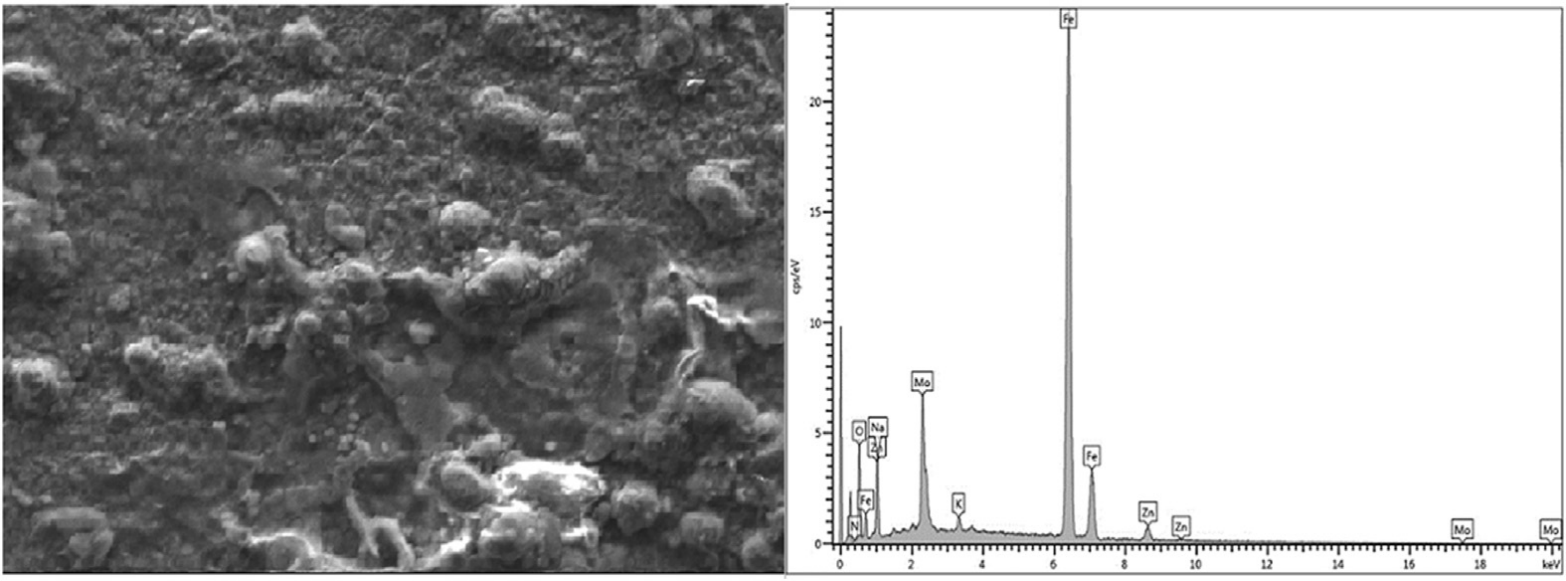
SEM/EDS spectra showing the surface morphology of Zn–Ni–10NbO_2_–1.0V deposition at mag ×1000.

**Fig. 3:**
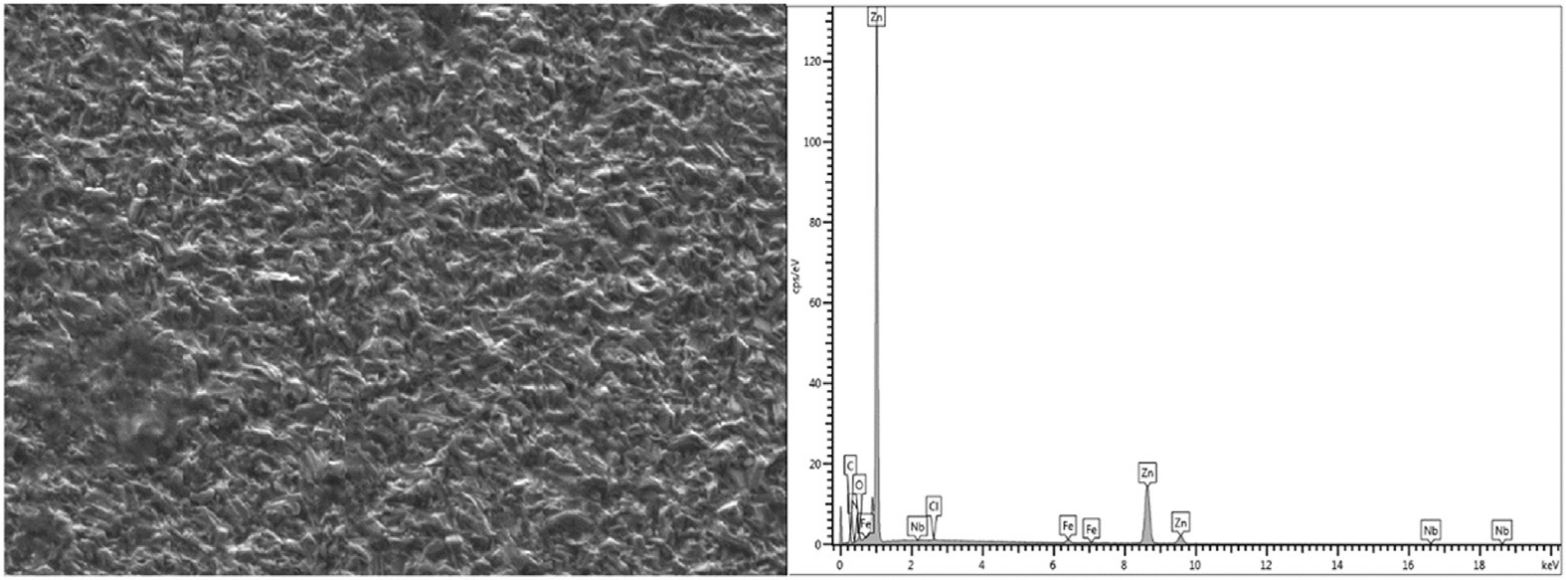
SEM/EDS spectra showing the surface morphology of Zn–Ni–15NbO_2_–1.0V deposition at mag ×1000.

**Fig. 4:**
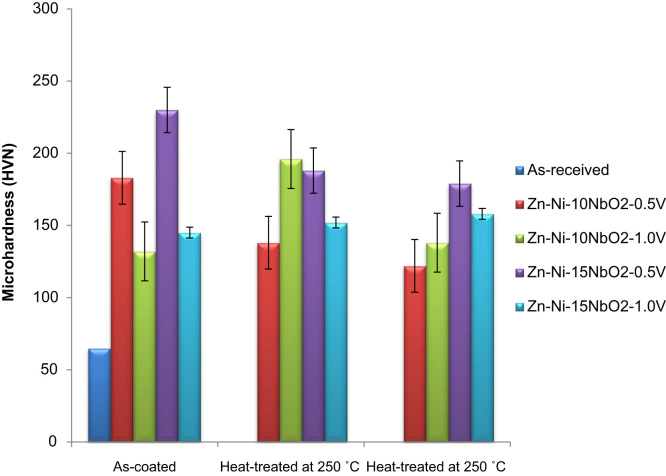
Microhardness chart variations for as-coated Zn–Ni–NbO_2_ deposited matrix plotted against the microhardness obtained after heat-treated at 250 °C and 350 °C.

**Fig. 5:**
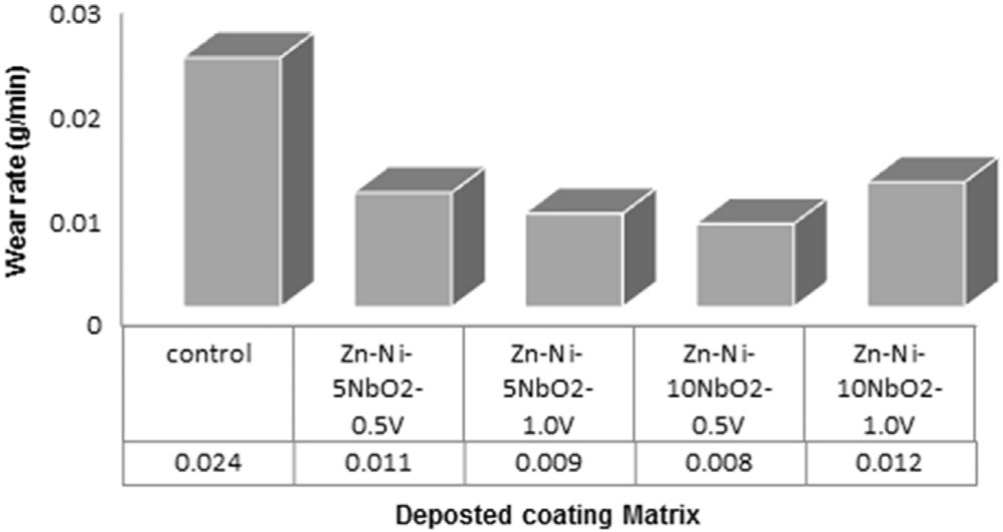
Variation of wear rate with time (Zn–Ni–NbO_2_ deposition).

Locally sourced mild steel sheet was sectioned to dimensions of 40 mm × 30 cm × 2 mm while the Zinc sheets of 85 mm × 45 mm × 5 mm with 99.99% were prepared as anodes. The sectioned mild steel specimens were prepared by polishing and grinding using successive grades of silicon carbide paper grit. The chemical composition of the mild steel substrate obtained from spectrometer analyzer as shown in Table 2. Analytical grade chemicals and distilled water were used to prepare the plating solutions with compositions and parameters shown in Table 1. The formulated solutions were then heated to 35 °C for easy admix and dissolution of any agglomerates in the bath solution as described by [1], [2], [3]. The electrolytic deposition of Zn–Ni–NbO_2_ deposition fabricated alloy coatings was performed in a single cell containing two zinc anodes and a single cathode at a time [4], [5]. The set up was done such that the distance between the anode and the cathode was kept at 10 mm (see Table 3). The prepared cathode and anodes were connected to the D.C. power supply through conducting wires. Applied voltage between 0.5 and 1.0 V was set to run for 20 min in order to successfully produce desired deposited specimens [6]. The results of the variation were seen in Table 4. Morphological, phase orientation, wear and micro-hardness characterizations were done to further investigate the produced deposits and the outcome presented in Fig. 1, Fig. 2, Fig. 3, Fig. 4, Fig. 5.

**Table 2:** Chemical Composition data of as-received mild steel.

Element	Fe	C	Mn	Si	P	S	Al	Ni
**% Content**	99.166	0.15	0.45	0.18	0.01	0.031	0.005	0.008

**Table 3:** Electrodeposition parameters data for Zn-Ni-NbO_2_ deposition.

**Sample**	**Time (min)**	**Voltage (V)**	**Additive concentration**
Zn–Ni–NbO_2_	20	0.5	10
Zn–Ni–NbO_2_	20	1.0	10
Zn–Ni–NbO_2_	20	0.5	15
Zn–Ni–NbO_2_	20	1.0	15

**Table 4:** Parameters variations data showing coating efficiency vs corrosion rate of Zn-Ni-NbO_2_ deposition.

**Sample**	**Corrosion rate (mm/yr)**	**Coating efficiency (%)**	**Surface coverage**	**Coating thickness (µm)**	**Weight gain**	**Coating per unit area (mg/mm**^**2**^**)**
Control	442.94	–	–	–	–	–
Zn–Ni–10NbO_2_	79,60	82,03	0,82	2961	0,0623	0,025979167
Zn–Ni–10NbO_2_	28,79	93,50	0,94	2803,5	0,5132	0,213833333
Zn–Ni–15NbO_2_	3,53	99,20	0,99	2869,5	0,2330	0,097083333
Zn–Ni–15NbO_2_	27,48	93,80	0,94	2605	0,2849	0,118704167
