# Supplementary material for: Data analysis and study of the influence of deposition power on the microstructural evolution and functionality of metallic phase composite coating
Source: Data Brief. 2018 Feb 7;17:757–62. doi: 10.1016/j.dib.2018.02.007 (PMC5988500; doi:10.1016/j.dib.2018.02.007)
Supplement: Supplementary file 1 — Transparency document [file mmc1.docx]

***COVER LETTER/CONFLICT OF INTEREST ATTESTATION***

*29th December, 2017*

*The Editor-in-Chief*

*Data in Brief*

***Subject:***  ***NO CONFLICT OF INTEREST***

*Dear Sir,*

*This serve to notify you that the manuscript is original of the authors work and there is no conflict of interest of any kind regarding the manuscript Data analysis and study of the influence of deposition power on the microstructural evolution and functionality of metallic phase composite coating*

*Sincerely yours,*

*Dr. OSI Fayomi*

*Department of Mechanical Engineering*

*Covenant University
Ota,*

*Nigeria*
